# Supplementary material for: Site selection for subtropical thicket restoration: mapping cold-air pooling in the South African sub-escarpment lowlands
Source: PeerJ. 2020 Apr 23;8:e8980. doi: 10.7717/peerj.8980 (PMC7183751; doi:10.7717/peerj.8980)
Supplement: Supplemental Information 1 [file peerj-08-8980-s001.docx]

**Supplementary Information. Descriptions of the site used for model calibration and filtering of thicket wide plots.**

***Data and observations of the study site used to for model calibration***

At Klipfontein (Fig. 4a), the mapped extent of different frost-risk areas (frost-free or frost-prone) were compared with the observed extent of the position of the intact biome boundary between thicket and Karoo shrubland vegetation — this boundary has been established to be frost driven by temperature logging and visual observations (Duker et al. 2015a, 2015b). In this valley, the Karoo vegetation is found exclusively in the zone of higher frost risk (slope <10°), and where there is a sharp change in gradient (occurring over approximately 25 m) on the north-facing slopes, thicket vegetation immediately becomes dominant. Extensive frost occurs in this valley, and coincides with the boundary of the thicket and Karoo vegetation, which coincides with the uppermost boundary of the high frost-risk zone.

Field observations indicate that the lowermost thicket clumps along this boundary are occasionally damaged by extreme frost events, especially where clumps are found within the high-risk zone, but those found only a short distance upslope in the low-risk zone appear to be unaffected. This appears to be due to a combination of factors, specifically that frost only occurs on relatively flat ground (slopes <10°) at Kaboega, and that these clumps are dominated by species that have higher levels of freezing tolerance than those that are visually more abundant on steeper slopes (>10°). The Klipfontein valley has two topographic features that facilitate cold air pooling and frost: 1) a far greater diameter than depth, and 2) a wide and very flat valley floor (Barr and Orgill 1989, Neff and King 1989, Lindkvist et al. 2000, Chung et al. 2006, Lundquist et al. 2008). This valley also has steep slopes without any flat areas at higher elevations that may trap cold air, such as those at the Buffels Nek valley (Fig. 4b, see below for further discussion), meaning that a very well defined vertical temperature gradient is likely to form on still and cloudless nights here, with cold air concentrating in the most low-lying areas (Lindkvist et al. 2000, Bigg et al. 2014).

At Buffels Nek, the boundary between high and low frost-risk areas also closely coincides with a relatively sharp increase in the angle of slope (Fig. 4b), although here it is slightly more gradual than at Klipfontein (Fig. 4a), occurring over approximately 50m. Above this transitional zone, there is an observably higher density of thicket clumps, indicating the start of the zone of degraded thicket (Fig. 4b). The flatter ground below this change in gradient is almost completely dominated by Karoo shrubs, except for certain frost refugia such as rocky outcrops, where the more frost tolerant thicket species persist, and in deep gullies such as those surrounding the main drainage line, where winter deciduous *Vachellia karroo* forms a woodland that may also provide some protection from frost. It has been previously found that cuttings of spekboom were severely damaged by frost that occurred below this change in gradient, with very low levels of survival by the end of winter (Duker et al. 2015a). The thicket clumps found below the frost line at Buffels Nek are, in general, dominated by more frost tolerant species, similar to the clumps found along the frost line at Klipfontein (see Duker et al. 2015a, 2015b). Similar to what we have observed at Klipfontein, the few individuals of less tolerant species (such as spekboom) that occur here do sustain severe frost damage during cold periods, and are geographically limited to sheltered microclimates such as dense bush clumps and rocky outcrops (Duker et al. 2015a, 2015b). This suggests that the pre-pastoral location of the biome boundary between thicket and Karoo shrubland vegetation in this valley was situated approximately where this change in gradient results in the occurrence of the ‘frost line’.

***Filtering of Thicket Wide Plot***

Plot data were collected for van der Vyver’s study, and he supplied a subset dataset of 227 out of the initial 300 plots; not all of the original 300 plots were usable for various reasons, such as landowners using the fenced plot as a livestock enclosure, fences being removed or stolen, or because of improper data collection (van der Vyver, 2018). A variety of factors are suggested to have influenced spekboom survival and plant height across the thicket wide plots, including herbivory, aspect, slope, surrounding vegetation and frost damage. Apart from observations of frost damage on plants, van der Vyver (2018) did not explore whether low temperatures and/or frost — likely driven by cold air pooling — had influenced the spekboom planted in the Thicket-Wide Plots.

At each of the 227 thicket-wide plots, we manually calculated the unique valley radius to generate the cold-air pool (i.e. frost-risk) maps. Calculating the radius for some plots was straightforward, where a plot was situated in a clearly defined valley, i.e. in a region of high topographic complexity (such as the valleys occurring along the Cape Folded Belt). However, the radii for most plots were problematic to estimate as these plots were situated in flat areas (within a radius of >5 km), or were not in a valley but on an inselberg or isolated mountain outside of areas that we felt were likely to be prone to cold air pooling. There were 135 plots that fell into this topographically inappropriate category and these were removed from the analysis to ensure that the analysis was limited to topographic regions prone to cold air pooling (i.e. clearly definable valleys) as the Lundquist et al. (2008) model is based on valley features.

In addition, the upper escarpment and interior plateau of southern Africa is prone to very low mean and minimum temperatures, and regular frost occurrence. Here we have also observed frost to occur in different areas of the landscape and under a broader set of topographic conditions (steeper slopes, on flat plateau and in slightly convex areas) than it does in the generally warmer and lower elevation sub-escarpment lowlands. We excluded all plots that occurred along the Great Escarpment or just south of it (above approximately 700m elevation), especially those occurring in flatter areas.

This left 92 plots in total, most of which were located in the Cape Fold Mountains of the sub-escarpment coastal plain. These plots were used to assess whether the frost model predictions could identify plots with lower NPP rates (t.ha^-1^.yr^-1^), determined using Mann-Whitney *U-*tests. Estimated browse intensity (0–4) was also included as part of the dataset provided (van der Vyver, 2018). We found that plots with high browse intensity (a visually estimated rating of 0-4) had significantly lower NPP rates than plots with lower browse intensities (1–3, p<0.001; determined with Mann-Whitney *U-*tests; data not shown), and thus we also removed these 22 plots from the analysis. In total, 70 less-browsed plots of the TWP dataset were used to test the accuracy of the frost model, with 40 plots found inside frost-free areas and 30 plots found in frost-prone areas.

***Site level phytomass accumulation rate calculation***

Aboveground phytomass was calculated from the following allometric model (van der Vyver, 2018):

NPP *_P.afra_* = 8.407 × 10^−5^ × (π*Cr* 2 *Hgt*) 0.978 × *DW _ratio_* × *C* *_frac_*

where phytomass for each tree (kg) = phytomass (kg); *Cr* = canopy radius measured as half the mean of the canopy diameter measurements taken at right angles to each other (cm); *Hgt* = maximum height of the plant (cm); *DW_ratio_* = dry:wet ratio of 0.271; *C _frac_* = fraction of carbon dry woody biomass (0.5; see MacDicken 1997).

The TWP treatment C (30cm tall truncheons planted 2m apart, at depths of 15cm) plot-level phytomass accumulation rate was calculated by dividing mean aboveground NPP per row of spekboom cuttings by the age of the particular restoration plot, measured from the date of planting to the date of sampling (months), and then extrapolating the result to metric tons phytomass per hectare per year (t.C.ha^−1^.yr^−1^).


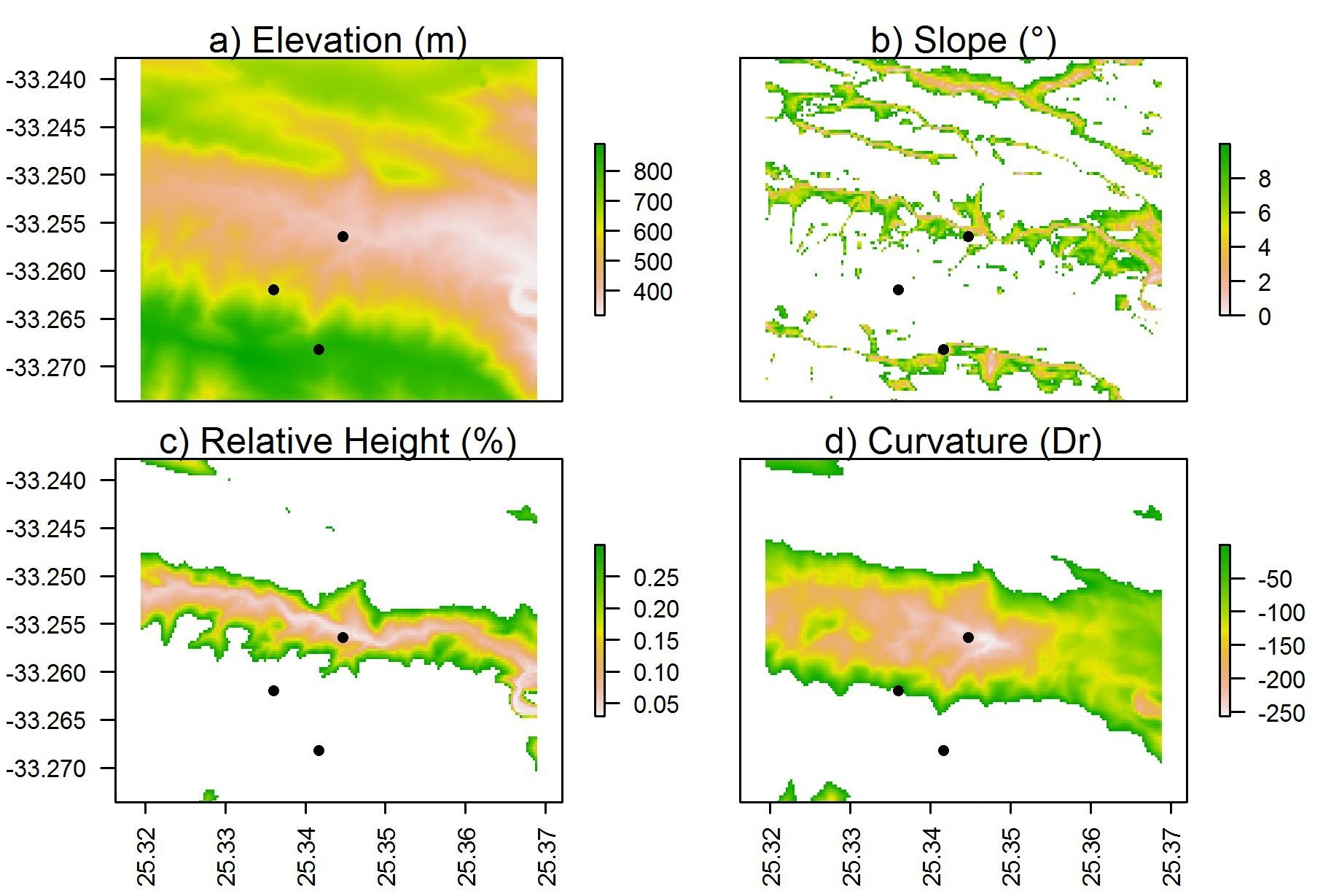


Fig. S1. Maps of a) elevation (m), b) slope (°), c) height relative to surroundings (%), and d) curvature (Dr; radians) for the Buffels Nek valley in the western portion of the Kaboega study site. Points indicate position of temperature loggers in the landscape described in Duker et al. 2015b. Cells unlikely to be prone to cold air pooling and frost are excluded. See text for explanation.


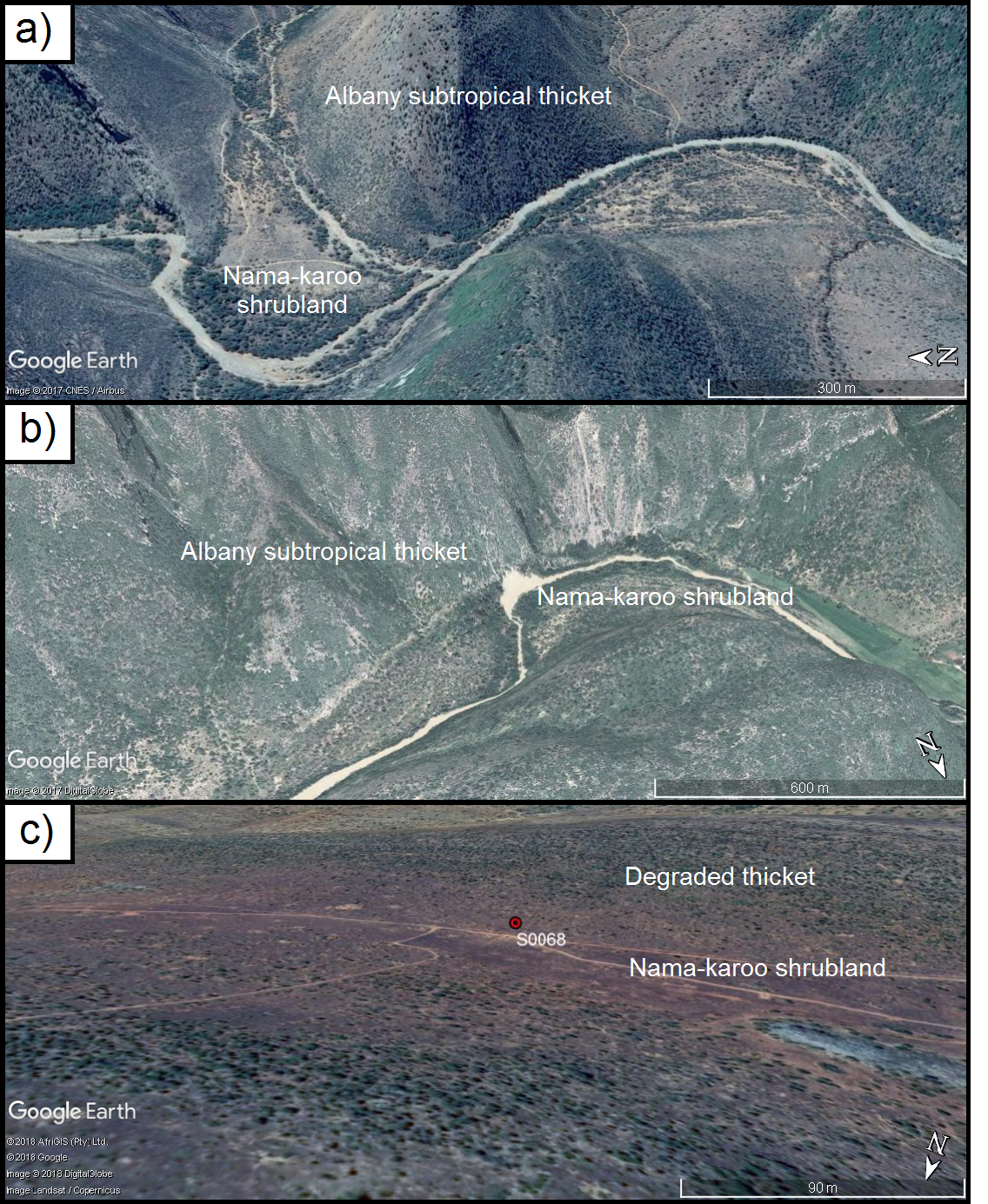


Fig. S2. The position of the three different model testing sites a) approximately 14km E of Steytlerville (testing site A: E24°29'39.69"; S33°18'59.68"), b) approximately 31km WNW of Kirkwood (testing site B: E25° 8'16.60"; S33°16'58.61") and c) approximately 22km W of Grahamstown (testing site C: E26°22'17.43"; S33°11'47.18”). Red line in c) indicates the valley diameter measurement included here for display purposes.
